# Reptarenavirus S Segment RNA Levels Correlate with the Presence of Inclusion Bodies and the Number of L Segments in Snakes with Reptarenavirus Infection—Lessons Learned from a Large Breeding Colony

**DOI:** 10.1128/spectrum.05065-22

**Published:** 2023-05-22

**Authors:** Tanja Thiele, Francesca Baggio, Barbara Prähauser, Andres Ruiz Subira, Eleni Michalopoulou, Anja Kipar, Udo Hetzel, Jussi Hepojoki

**Affiliations:** a Institute of Veterinary Pathology, Vetsuisse Faculty, University of Zurich, Zurich, Switzerland; b Center for Clinical Studies, Vetsuisse Faculty, University of Zurich, Zurich, Switzerland; c Department of Veterinary Biosciences, Faculty of Veterinary Medicine, University of Helsinki, Helsinki, Finland; d Department of Virology, Medicum, Faculty of Medicine, University of Helsinki, Helsinki, Finland; National Chung Hsing University

**Keywords:** arenavirus, boid inclusion body disease, coinfection

## Abstract

Reptarenaviruses cause boid inclusion body disease (BIBD), a fatal disease particularly impacting captive boa constrictor collections. The development of cytoplasmic inclusion bodies (IBs) comprising reptarenavirus nucleoprotein (NP) in many cell types of affected snakes is characteristic of BIBD. However, snakes can harbor reptarenaviruses without showing IBs, hence representing carriers and a potential source of transmission. The RNA genome of reptarenaviruses comprises a small (S) and a large (L) segment, and the snakes with BIBD commonly carry a swarm of reptarenavirus segments. To design sensitive and reliable tools for the diagnosis of reptarenavirus infection in snake colonies, we used metatranscriptomics to determine the reptarenavirus segments present in a large boa constrictor breeding colony. The analysis identified one reptarenavirus S segment and three L segments in the colony. The sequence data served to design real-time reverse transcription-PCR (RT-PCR) targeting the found S segment. This allowed us to identify all infected animals and to quantify the S segment RNA levels, which we found to correlate with the presence of IBs. We further found a positive correlation between the number of L segments and the S segment RNA level, which could suggest that L segment excess also contributes to the IB formation. Information on cohousing of the snakes showed a clear association of reptarenavirus infection with cohousing in general and cohousing with infected animals. Information on breeding and offspring confirmed that vertical transmission occurred. Furthermore, our data suggest that some animals might be able to clear the infection or at least exhibit transient or intermittent viremia.

**IMPORTANCE** Boid inclusion body disease (BIBD) is caused by reptarenavirus infection, and while reptarenavirus nucleoprotein is the main component of the inclusion bodies (IBs) characteristic of BIBD, not all reptarenavirus-infected snakes demonstrate IBs in their cells. Identification of infected individuals is critical for controlling the spread of the disease; however, the genetic divergence of reptarenaviruses complicates reverse transcription-PCR (RT-PCR)-based diagnostics. Here, we tested a next-generation-sequencing-based approach to establish a tailored “colony-specific” set of diagnostic tools for the detection of reptarenavirus small (S) and large (L) genome segments. With this approach, we could demonstrate that an S-segment-specific RT-PCR is highly effective in identifying the infected individuals. We further found the S segment RNA level to positively correlate with the presence of IBs and the number of L segments, which could direct future studies to identify the BIBD pathogenetic mechanisms.

## INTRODUCTION

Boid inclusion body disease (BIBD) is a widely distributed transmissible disease of constrictor snakes named after the formation of characteristic intracytoplasmic inclusion bodies (IBs) in many cell types in boas (1–4). Descriptions of the disease in captive snakes of the families *Boidae* and *Pythonidae*, several genera of which are susceptible (2, 3, 5, 6), go back as far as the 1970s (1, 7), decades before reptarenaviruses were identified as the causative agent (3, 4, 8–10). Recently, we have shown that BIBD occurs also in wild boa constrictors, where we could confirm cases dated back to 1989 (11). This and the fact that we also identified healthy wild snakes with reptarenavirus infection indicate that the viruses causing BIBD circulate in the wild and further suggest that they may have entered captive collections through trading of wild-caught animals (11).

BIBD is most commonly observed in boas, where it takes a variable clinical course that ranges from subclinical to a disease with neurological signs, such as opisthotonos and head tremor as well as disorientation, regurgitation, and anorexia (1, 2, 12). Affected boas may develop secondary bacterial, fungal, or protozoal infections that often manifest as pneumonia, stomatitis, and/or systemic disease (1, 2, 7), which could indicate immunosuppression as one of BIBD’s secondary manifestations. Severe secondary infections were also responsible for the most overt pathological changes observed in affected indigenous snakes that we have recently identified in both Brazil and Costa Rica (11, 13).

The family *Arenaviridae* in the order *Bunyavirales* is at present divided into four genera, *Mammarenavirus*, *Reptarenavirus*, *Hartmanivirus*, and *Antennavirus* (14, 15), but according to a recent proposal the family could in the future include a fifth member, the genus *Innmovirus* (https://ictv.global/filebrowser/download/10881). The bisegmented reptarenavirus genome is a single-stranded negative-sense RNA with ambisense coding strategy (15). The small (S) segment encodes the nucleoprotein (NP) and the glycoprotein precursor (GPC), whereas the large (L) segment encodes the zinc finger matrix Z protein (ZP) and the RNA-dependent polymerase (RdRp) (15). Several reports have shown that snakes with BIBD often carry several reptarenavirus L and S segments, and commonly more L than S segments are found in the positive animals (11, 13, 16–18). Animals with BIBD often harbor hartmaniviruses as well, but so far, there is no evidence to suggest that they would contribute to the BIBD pathogenesis (19, 20). Infected boa constrictors shed reptarenavirus RNA with skin, feces, and urates, indicating these as the potential sources of horizontal transmission (9, 18). However, the viruses are also transmitted vertically (17). The transmissible nature of BIBD and its dire consequences, i.e., the recommendation to euthanize affected animals (1) in breeding colonies and zoological snake collections (2, 4), call for options to control its spread and to monitor colonies for the presence of reptarenaviruses.

In boas, the detection of IBs in histological specimens such as liver biopsy specimens or in blood cells using blood smears presents the antemortem diagnostic gold standard for BIBD (2–4, 7, 17, 20–22). However, studies in both naturally and experimentally infected snakes have provided strong evidence that reptarenavirus infection does not necessarily directly result in BIBD, i.e., that infected snakes can remain without IBs and clinically healthy for months or even years (3, 5, 9, 10, 20, 22, 23). We thus propose three categories: reptarenavirus infection (no IBs, i.e., IB negative), nonclinical BIBD (IB positive but no clinical disease), and clinical BIBD (IB positive and clinical disease) to further classify the disease. Snakes with nonclinical BIBD and asymptomatic reptarenavirus carriers represent an unidentified source of infection for other animals, introducing, spreading, and maintaining the viruses in colonies. It is such clinically healthy carriers that make screening of colonies for the presence of reptarenaviruses important. However, the large genetic variability among reptarenaviruses poses a substantial challenge for the establishment of reverse transcription-PCR (RT-PCR)-based diagnostic tools.

The present study reports an attempt toward a feasible diagnostic approach for large breeding colonies. To that end, we made use of blood samples from 183 clinically healthy boa constrictors (Boa constrictor imperator) of a European breeding colony collected at one sampling time point with the aim to screen the colony for BIBD and reptarenavirus infection. We examined the collected samples for the presence of cytoplasmic IBs (diagnosis of BIBD) in blood cells and then performed a metatranscriptomic analysis to identify the dominating reptarenaviruses and the “reptarenavirome” of the colony. The metatranscriptomic analysis identified only a single S segment, i.e., University of Giessen virus (UGV) or S6 according to reference 18, which served to design a specific quantitative reverse transcription-PCR (qRT-PCR) that enabled screening of all individual animals for reptarenavirus infection. In addition, we studied infected animals for the prevalence of the three L segments identified by the metatranscriptomic approach and in a further step for the presence of L segments found in our earlier studies. This identified two more L segments which were also assessed for their distribution among infected animals. We subsequently investigated the cohort for several population and risk parameters and the association of UGV S segment RNA levels and the number of L segments with the presence of IBs.

## RESULTS

### Diagnosis of BIBD in the study cohort and its association with population parameters.

**FIG 1:**
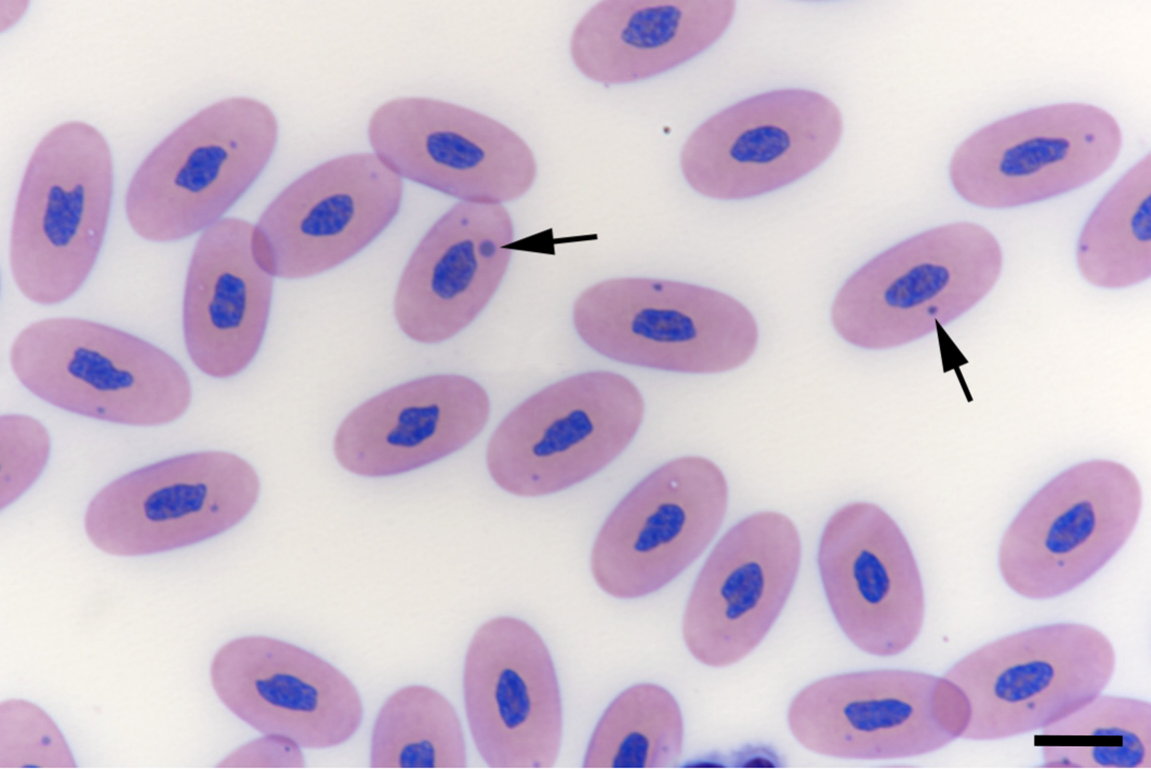
Blood smear, snake with nonclinical BIBD (animal 97). Several erythrocytes exhibit variably sized cytoplasmic inclusion bodies (IBs) (arrows). May-Grünwald-Giemsa stain. Bar, 5 μm.

**FIG 2:**
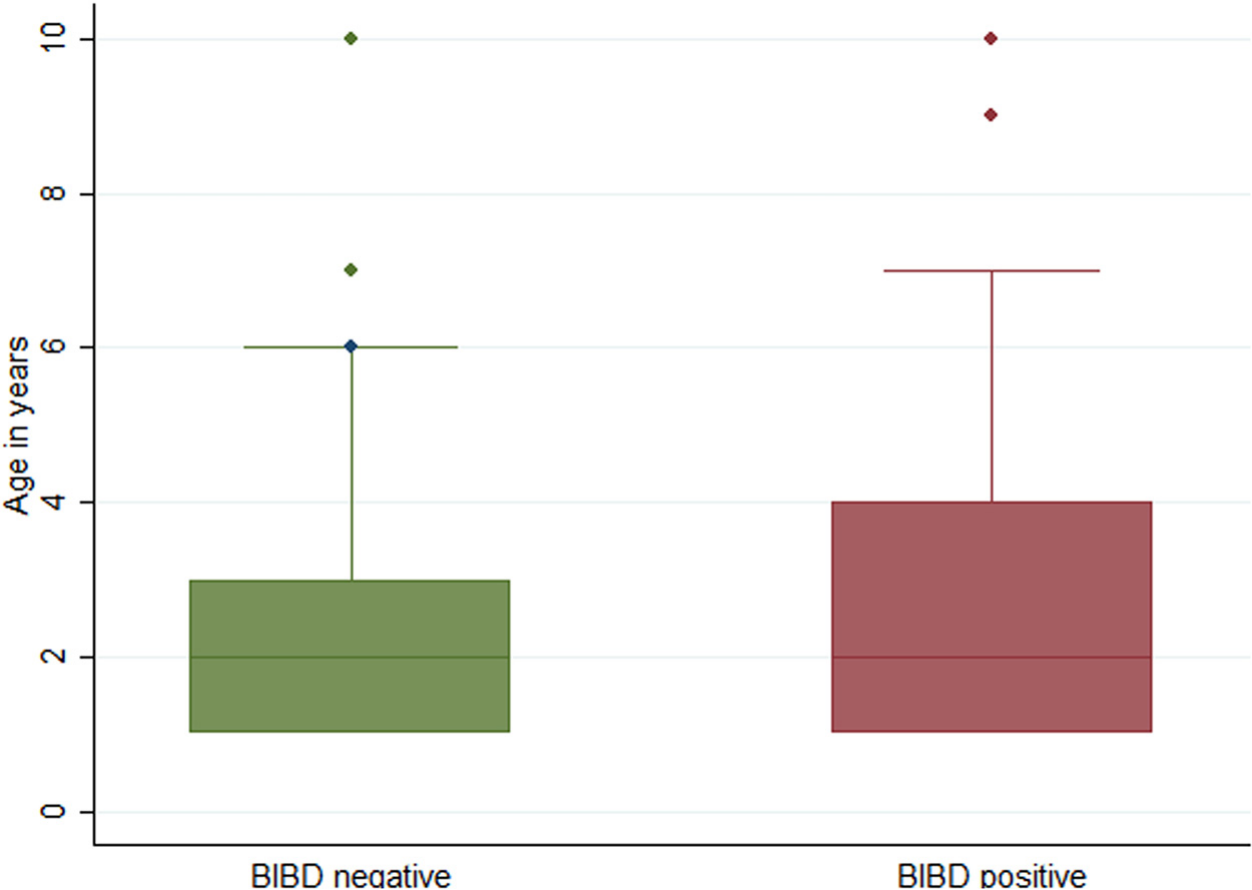
Box-and-whisker plot showing the age distribution of the snakes in the colony that were diagnosed as BIBD negative (no evidence of IBs in blood cells) and as BIBD positive (presence of IBs in blood cells) by cytological examination of a May-Grünwald-Giemsa-stained blood smear. Note that all tested animals were clinically healthy; hence, the IB-positive snakes were diagnosed with nonclinical BIBD.

The detection of characteristic IBs within blood cells in blood smears presents the diagnostic gold standard of BIBD (2–4, 20–22, 24). We thus examined May-Grünwald-Giemsa-stained blood smears from all animals for the presence of IBs (Fig. 1) and found 28 of the 183 animals (15.3%) to be IB (i.e., BIBD) positive. Table 1 summarizes the sex distribution for both BIBD-positive and BIBD-negative animals. Animal age ranged from 1 to 10 years, with a median of 2 years. Figure 2 summarizes the age distribution of BIBD-negative and -positive animals. There was no significant association between the detection of IBs and the age (*z* = 0.482, *P* = 0.6298) or sex (χ^2^ = 0.8107, *P* = 0.368) of the snakes.

**TABLE 1:** BIBD status (determined by the presence of cytoplasmic inclusion bodies in blood cells in a May-Grünwald-Giemsa-stained blood smear), infection status (determined by qRT-PCR for UGV-1), and sex distribution of the examined snakes

Characteristic	No. (%) of samples with status type:		
	Negative	Positive	Total
	BIBD status^*a*^		
Sex			
Male	97 (89.61)	13 (13.39)	112
Female	58 (81.69)	15 (18.31)	71
Total	155 (84.70)	28 (15.30)	183
	Infection status		
BIBD status			
Negative	146 (94.19)	9 (5.81)	155
Positive	0 (100)	28 (100)	28
Total	146 (79.78)	37 (20.22)	183
	Infection status^*b*^		
Sex			
Male	93 (83.04)	19 (16.96)	112
Female	53 (74.65)	18 (25.35)	71
Total	146 (79.78)	37 (25.35)	183

a No significant association between BIBD status and sex distribution. For negative versus positive, the χ^2^ value is 0.8107 and the *P* value is 0.368.

b No significant association between infection status and sex distribution. For negative versus positive, the χ^2^ value is 1.895 and the *P* value is 0.169.

### Identification and analysis of the breeding collection’s reptarenavirome.

Snakes with BIBD often carry a variety of reptarenavirus L and S segments (16, 18), and according to our earlier studies snake colonies may harbor various reptarenaviromes, i.e., specific compilations of L and S segments (13, 17). This led us to speculate that performing a metatranscriptomic study on a subpopulation of snakes in a given collection would enable generation of collection-specific tools for screening of the animals. We thus selected a set of 15 blood samples (seven BIBD positive [with IBs in blood cells; IB positive] and eight BIBD negative [no evidence of IBs in blood cells; IB negative]) and analyzed these by next-generation sequencing (NGS). We utilized Lazypipe (25) for the data analysis, which allowed us, similarly to earlier studies (11, 16, 17), to obtain some full-length reptarenavirus and hartmanivirus S and L segments. Reptarenavirus segments were detected in the IB-positive samples (samples 20, 56, 97, 103, 117, 170, and 180), of which three also harbored hartmanivirus segments (samples 56, 170, and 180). In three of the IB-negative samples (samples 13, 34, and 50), hartmanivirus segments but no reptarenaviruses were detected. The remaining five IB-negative animals did not carry segments of either virus (samples 86, 87, 98, 104, and 155).

More specifically, we identified the following reptarenavirus segments: University of Giessen virus (UGV, or S6 according to the nomenclature of Stenglein et al. [18]) S segment and the L segments of aurora borealis virus 3 (ABV-3, L3), tavallinen suomalainen mies virus 1 (TSMV-1, L7), and keijut pohjoismaissa virus 1 (KePV-1, L15). The hartmanivirus segments identified included the S and L segments of veterinary pathology Zurich virus (VPZV) and the S segment of old schoolhouse virus 1 (OScV-1). Since the results of a previous study indicated that the presence of hartmaniviruses in the samples does not correlate with the presence of IBs (20), we did not analyze the hartmanivirus segments further. Reference assembly using all of the identified reptarenavirus S and L segments then served to confirm the presence of the identified segments in each of the samples. The NGS results are summarized in Table 2 and show the segment identified in each of the studied samples as well as the number of reads matching each segment. L-segment-specific RT-PCR further served to confirm the reference assembly results. The results of this approach for the animals testing positive by UGV/S6 S segment qRT-PCR are provided in Table S1 in the supplemental material.

**TABLE 2:** Reference assembly of the samples subjected to NGS^*b*^

Sample no.	BIBD status^*a*^	NGS result; closest known arenavirus segment	Reference assembly template (length in nt)^*c*^	No. of reads mapped	Avg coverage (fold)	Coverage range (fold)
20	+	L3 (KP071572.1)	UGV/S6 (3,433)	2,115	196	2–787
		TSMV-1 (L, MH483060.1)	ABV-3/L3 (6,903)	441	26	1–74
			TSMV-1/L7 (6,838)	574	32	1–135
			KePV-1/L15 (6,783)	None		
56	+	UGV-1 (S, MH483061.1)	UGV/S6 (3,433)	31,341	2,060	1–7,627
		L3 (KP071629.1)	ABV-3/L3 (6,903)	6,718	242	1–579
		L7 (KP071544.1)	TSMV-1/L7 (6,838)	7,025	234	3–837
		OScV-1 (S, MH483028.1)	KePV-1/L15 (6,783)	None		
97	+	UGV-1 (S, NC_039005.1)	UGV/S6 (3,433)	3,891	373	2–1,717
		ABV-3 (L, KR870025.1)	ABV-3/L3 (6,903)	845	55	1–181
		TSMV-1 (L, KR870026.1)	TSMV-1/L7 (6,838)	991	50	1–206
			KePV-1/L15 (6,783)	None		
103	+	S6 (KP071588.1)	UGV/S6 (3,433)	7,209	624	5–2,941
		ABV-3 (L, KR870025.1)	ABV-3/L3 (6,903)	1,667	78	1–259
		L7 (KP071590.1)	TSMV-1/L7 (6,838)	1,805	78	1–299
			KePV-1/L15 (6,783)	None		
117	+	UGV-1 (S, MH483052.1)	UGV/S6 (3,433)	10,192	809	1–3,490
		L3 (KP071629.1)	ABV-3/L3 (6,903)	4,379	223	1–839
		KePV-1 (L, MH483066.1)	TSMV-1/L7 (6,838)	None		
			KePV-1/L15 (6,783)	1,849	90	1–384
170	+	UGV-1 (S, MH483061.1)	UGV/S6 (3,433)	4,296	341	3–1,397
		ABV-3 (L, MH483045.1)	ABV-3/L3 (6,903)	2,878	148	2–512
		VPZV-1 (L, MH483036.1)	TSMV-1/L7 (6,838)	None		
			KePV-1/L15 (6,783)	None		
180	+	UGV-1 (S, MH483061.1)	UGV/S6 (3,433)	5,683	459	1–2,035
		L3 (KP071596.1)	ABV-3/L3 (6,903)	3,300	174	1–632
		VPZV-2 (S, MH483043.1)	TSMV-1/L7 (6,838)	None		
			KePV-1/L15 (6,783)	None		
13	−	OScV-1 (S, MH483024.1)	UGV/S6 (3,433)	None		
			ABV-3/L3 (6,903)	None		
			TSMV-1/L7 (6,838)	None		
			KePV-1/L15 (6,783)	None		
34	−	VPZV-2 (S, MH483043.1)	UGV/S6 (3,433)	None		
			ABV-3/L3 (6,903)	None		
			TSMV-1/L7 (6,838)	None		
			KePV-1/L15 (6,783)	None		
50	−	VPZV-1 (L, MH483044.1)	UGV/S6 (3,433)	None		
		VPZV-2 (S, MH483043.1)	ABV-3/L3 (6,903)	None		
			TSMV-1/L7 (6,838)	2	1.7	1–2
			KePV-1/L15 (6,783)	None		
86	−	Neg	UGV/S6 (3,433)	None		
			ABV-3/L3 (6,903)	None		
			TSMV-1/L7 (6,838)	None		
			KePV-1/L15 (6,783)	None		
87	−	Neg	UGV/S6 (3,433)	None		
			ABV-3/L3 (6,903)	None		
			TSMV-1/L7 (6,838)	None		
			KePV-1/L15 (6,783)	None		
98	−	Neg	UGV/S6 (3,433)	None		
			ABV-3/L3 (6,903)	None		
			TSMV-1/L7 (6,838)	None		
			KePV-1/L15 (6,783)	None		
104	−	Neg	UGV/S6 (3,433)	None		
			ABV-3/L3 (6,903)	None		
			TSMV-1/L7 (6,838)	None		
			KePV-1/L15 (6,783)	None		
155	−	Neg	UGV/S6 (3,433)	None		
			ABV-3/L3 (6,903)	None		
			TSMV-1/L7 (6,838)	None		
			KePV-1/L15 (6,783)	None		

a BIBD status was determined by the presence of cytoplasmic inclusion bodies in blood cells in a May-Grünwald-Giemsa-stained blood smear.

b OScV-1, old schoolhouse virus 1; TSMV-1, tavallinen suomalainen mies virus 1; VPZV, veterinary pathology Zurich virus; UGV-1, University of Giessen virus 1; ABV-3, aurora borealis virus 3; KePV-1, keijut pohjoismaissa virus 1; nt, nucleotides; Neg, negative.

c Sequences used in the reference assemblies: UGV-1, GenBank accession no. NC_039005 (length, 3,433 nt); L3, KP071602.1 (length, 6,903 nt); L7, KP071544.1 (length, 6,838 nt); KePV-1, MH483066.1 (length, 6,783 nt). Reference assemblies with SVaV-1 and KaBV-1 L segments did not yield any matching reads, and the segments are therefore not included in the table.

### Screening of the study cohort for reptarenavirus infection.

Since the NGS results provided strong evidence that the reptarenavirome in the colony comprised only one S segment, i.e., UGV, we screened all samples for its presence by qRT-PCR. Because the IBs mainly comprise reptarenavirus NP expressed from the S segment (3, 4, 21), we further hypothesized that quantification of UGV S segment levels might also serve to determine whether the BIBD status (i.e., the presence or absence of IBs in blood cells in a cytological specimen of a blood smear; IB positive/negative) is associated with differences in viral loads.

Of the 183 tested animals, 37 (20.2%) were found to be reptarenavirus infected, i.e., to carry the UGV S segment. These comprised all 28 IB-positive animals and nine of the 155 IB-negative animals (5.8%), representing 15.3% (28/183) and 4.9% (9/183) of the tested population, respectively (Table 1). Among the latter was also animal 13, in which our NGS approach had failed to detect a reptarenavirus (Table S1). Table 1 summarizes the sex distribution for both infected (UGV-1-positive) and uninfected (UGV-1-negative) animals.

### Correlation of S segment RNA levels and the presence of IBs.

Quantification of the UGV S segment RNA levels in the infected animals identified a substantial variation among the samples, ranging from fewer than 1 copy/ng total RNA to close to 10 million copies/ng total RNA in the blood (Table S1). For the statistical analyses, we applied square root transformation of the UGV S segment RNA levels obtained to achieve their normality.

**FIG 3:**
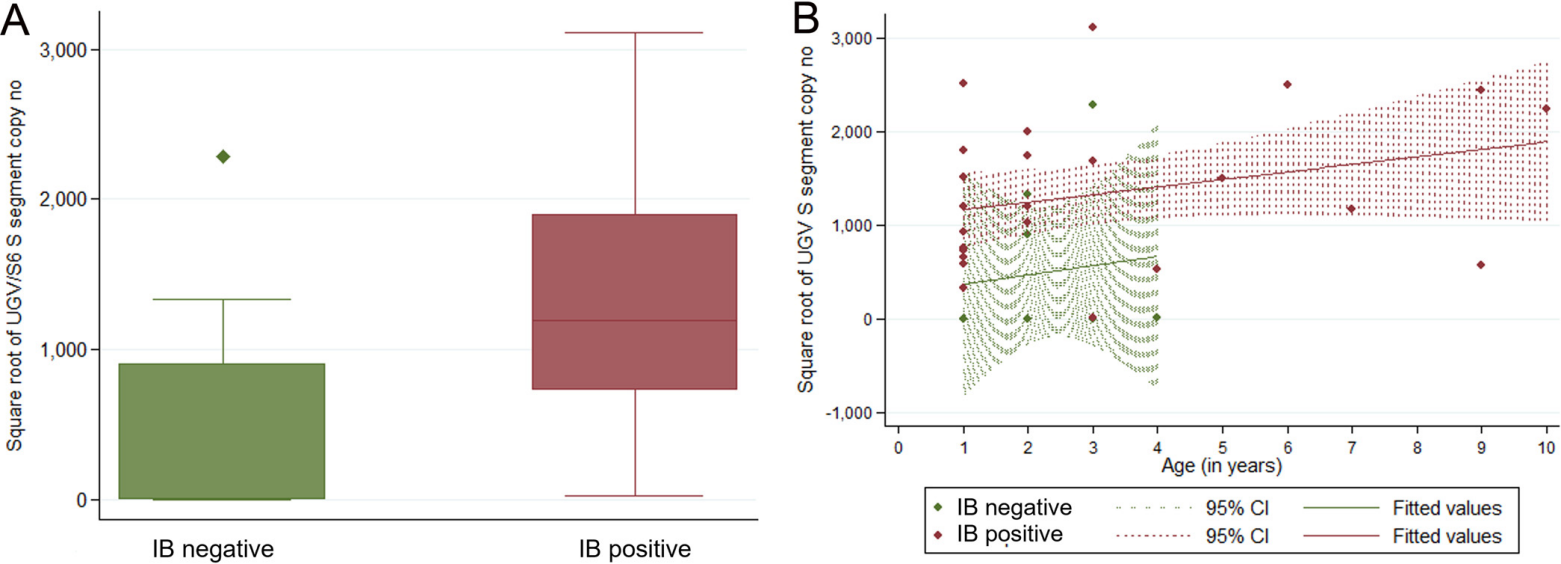
Illustration of the association between UGV S segment RNA levels (as the square root of the UGV/S6 S segment copies/nanogram of total RNA) and BIBD status (absence or presence of IBs in blood smear) (A) and age (B), respectively. (A) A box-and-whisker plot showing UGV S segment RNA levels in IB-negative and IB-positive animals. (B) A scatterplot and fitted lines with confidence intervals (CIs) of the association between UGV S segment RNA levels and age of the IB-negative and IB-positive animals.

A comparison of IB-positive and IB-negative animals showed significantly higher UGV RNA levels in animals with IBs (*t* = −2.8225, df = 35, *P* < 0.01) (Fig. 3A). However, each group contained outliers; three animals with reptarenavirus infection but no IBs (animals 35, 109, and 110) showed very high UGV S segment RNA levels, and one IB-positive animal (animal 5) had a substantially lower UGV RNA level than the other snakes in the group; all outliers were found among the adult animals, aged at least 3 years (Table S1). Including age in the analysis confirmed the association of higher UGV S segment RNA levels with the presence of IBs regardless of age (*P* < 0.05, *F* [1, 32] = 4.76, *R*^2^ = 0.2348) (Fig. 3B). However, there was no significant association between UGV RNA levels and age (*P* = 0.1, *F* [1, 32] = 2.91, *R*^2^ = 0.0547) or sex (*t* = 1.1914, df = 35, *P* = 0.2415).

Because the L segment encodes the RdRp responsible for replication and transcription of viral RNA, it is essential for completion of the replication cycle. Having observed considerable variation in the S segment RNA levels, we hypothesized that the number of L segments could contribute to the efficacy of S segment replication and transcription and thus to the level of S segment RNA as measured by qRT-PCR, which does not distinguish between genomic RNA, antigenomic RNA, or mRNA. To study the hypothesis, we analyzed the UGV/S6 S-segment-positive samples by RT-PCR for the presence of the three L segments that we had identified (ABV-3/L3, TSMV-1/L7, and KePV-1/L15). This initial analysis detected a significant association between the number of L segments and the presence of IBs, which prompted us to test a subset of UGV/S6 S segment RNA-positive samples (*n* = 12) for the presence of additional L segments that we had identified in our earlier studies (17). Indeed, primers targeting suri vanera viruses 1 and 2 (SVaV-1 and -2/L12) and Kaltenbach virus 1 (KaBV-1) yielded a PCR amplicon in some of the samples tested. We then screened the remaining UGV/S6 S-segment-positive samples for the presence of these segments and found 8 samples that amplified reptarenavirus L segment RNA with SVaV-1- or -2/L12-targeting primers and 10 samples with KaBV-1 L-targeting primers. Sanger sequencing of the RT-PCR products confirmed the presence of reptarenavirus L segments. Basic local alignment search tool (BLAST, https://blast.ncbi.nlm.nih.gov/Blast.cgi) analysis showed some (*n* = 2, sample 21 for SVaV-1 L and sample 89 for KaBV-1 L) of the amplicons to likely represent L segments that have not yet been characterized (nucleotide identity, <76%; sample 21, 72.5% identity to SVaV-1 L; sample 89, 72.99% identity to KaBV-1 L). The BLAST analysis showed the remaining 16 samples to contain an L segment of either SVaV or KaBV (not identical to SVaV-1 or -2 or to KaBV). Reference assembly from the NGS data using SVaV-1/L12 and KaBV-1 L segments as the reference did not identify any read in any of the samples analyzed by NGS, suggesting that the sequencing approach had identified all segments in the 15 animals studied. In addition, none of the SVaV or KaBV L-segment-positive animals had been included in the original metatranscriptomic analysis, thus increasing our confidence that the metatranscriptomic analysis had identified all segments in the samples analyzed.

Of the 37 samples tested, 35 (94.6%) were found to carry ABV-3/L3, 14 (37.8%) were found to carry TSMV-1/L7, 12 (32.4%) were found to carry KePV-1/L15, 8 (21.62%) were found to carry SVaV/L12, and 10 (27.03%) were found to carry the KaBV L segment. ABV-3/L3 was the L segment detected in all IB-negative snakes and in all IB-positive snakes with the exception of two animals (animals 21 and 120). These carried only TSMV-1/L7 and SVaV/L12 (animal 21) and the KePV-1/L15 (animal 120) L segment, respectively. ABV-3/L3 was the only L segment detected in the reptarenavirus-infected animals without IBs, except for the animal with the exceptionally high UGV/S6 segment RNA level (animal 35), which also carried the TSMV-1/L7 and SVaV/L12 L segments. In contrast, only two IB-positive snakes had ABV-3/L3 as the only L segment (animal 5, the snake with the very low UGV/S6 S segment RNA level, and animal 119).

Seven IB-positive animals were positive for both ABV-3/L3 and TSMV-1/L7 L segments (animals 20, 56, 57, 88, 97, 103, and 181), and another two were positive for ABV-3/L3 and KePV-1/L15 L segments (animals 117 and 118). Three and four L segments were detected only in IB-positive snakes with high UGV/S6 segment RNA levels. Specifically, three animals were positive for ABV-3/L3, KePV-1/L15, and KaBV L segments (animals 123, 124, and 179), two were positive for ABV-3/L3, TSMV-1/L7, and KaBV L segments (animals 19 and 89), one was positive for ABV-3/L3, TSMV-1/L7, and SVaV/L12 L segments (animal 35), and one was positive for ABV-3/L3, KePV-1/L15, and SVaV/L12 L segments (animal 116). Snakes carrying four L segments included three animals positive for ABV-3/L3, KePV-1/L15, SVaV/L12, and KaBV L segments (animals 121, 122, and 125), one positive for ABV-3/L3, TSMV-1/L7, SVaV/L12, and KaBV L segments (animal 44), one positive for ABV-3/L3, TSMV-1/L7, KePV-1/L15, and KaBV L segments (animal 58), and one positive for ABV-3/L3, TSMV-1/L7, KePV-1/L15, and SVaV/L12 (animal 59). We found no animal that carried all five L segments identified in the collection.

Individual animal data are presented in Table S1.

**FIG 4:**
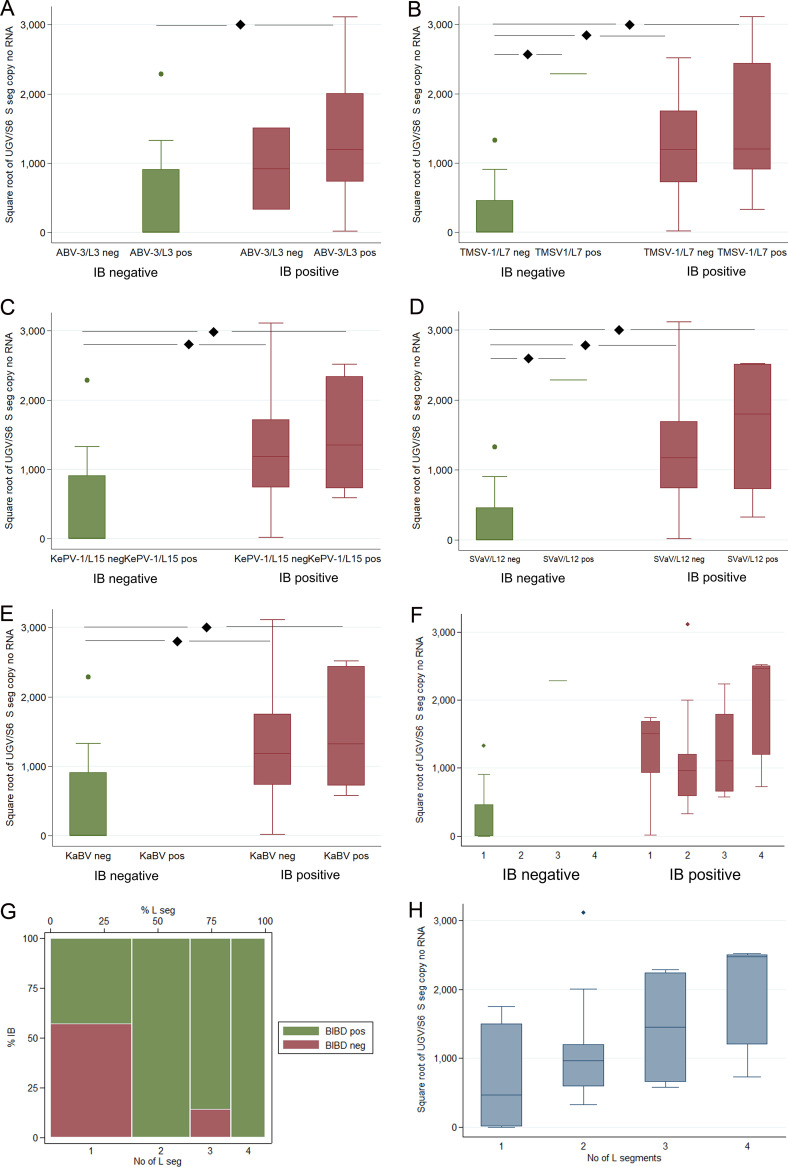
Box-and-whisker plots illustrating the association between the detection of the different reptarenavirus L segments, BIBD status (absence or presence of IBs in blood smear), and UGV S segment RNA levels (as the square root of the UGV/S6 S segment copies/nanogram of total RNA). (A) ABV-3/L3. (B) TSMV-1/L7. (C) KePV-1/L15. (D) SVaV/L12. (E) KaBV. (F) Association between the UGV/S6 S segment levels and the number of L segments in IB-negative and IB-positive animals. (G) Association between the UGV/S6 S segment levels and the number of L segments detected in the animals. (H) Association between the UGV/S6 S segment levels and the number of L segments regardless of the presence or absence of IBs. seg, segment; pos, positive; neg, negative.

The detection of the ABV-3/L3 L segment was not significantly associated with the presence of IBs (χ^2^ = 0.6796, *P* = 0.410) or the UGV/S6 S segment RNA levels (*t* = −0.3936, df = 35, *P* = 0.6963) (Fig. 4A; Tables S2 and S3). The detection of the TMSV-1/L7 L segment was not significantly associated with the presence of IBs (χ^2^ = 3.6118, *P* = 0.057) but was significantly associated with the UGV/S6 segment RNA level (*t* = −2.3924, df = 35, *P* < 0.05) (Fig. 4B; Tables S2 and S3). The detection of the KePV-1/L15 L segment was significantly associated with the presence of IBs (χ^2^ = 5.7086, *P* < 0.05) but not with the UGV/S6 segment RNA levels (*t* = −1.5377, df = 35, *P* = 0.1331) (Fig. 4C; Tables S2 and S3). The detection of the SVaV/L12 segment was not significantly associated with the presence of IBs but had a negative association with the levels of UGV/S6 segment RNA (*t* = −2.2013, df = 35, *P* < 0.05) (Fig. 4D; Tables S2 and S3). The detection of the KaBV L segment was significantly associated with the detection of IBs (χ^2^ = 4.4048, *P* < 0.05) but not with the levels of UGV/S6 segment RNA (*t* = −1.4962, df = 35, *P* = 0.1436) (Fig. 4E; Tables S2 and S3). Overall, in line with the significant association between UGV/S6 segment RNA levels and the presence of IBs (*n* = 37, *P* < 0.01, *R*^2^ = 0.2945) (Fig. 3A, Fig. 4F, and Table S2), there was a significant association between the number of L segments and the presence of IBs (χ^2^ = 10.00, *P* < 0.05) (Fig. 4F and G). The UGV/S6 S segment level appeared to increase together with the number of L segments (Fig. 4H). Pairwise comparison of means identified a significant association between the number of L segments and the level of UGV/S6 S segment RNA within the IB-negative animals between those with one L segment and those with three (*t* = 4.38, *P* < 0.005). There were no IB-negative animals with four L segments (Fig. 4F and G). There was no significant association between sex and the detection of ABV-3/L3, TMSV-1/L7, SVaV/L12, and KaBV L segments. There was a negative association between the detection of the KePV-1/L15 L segment and male animals (χ^2^ = 3.9759, *P* < 0.05). There was no significant association between the detection of L segments and age and between the detection of the different L segments except for a significant association between KePV-1/L15 and KaBV (χ^2^ = 8.8256, *P* < 0.005).

### Cohousing, breeding, and offspring of the cohort.

In the examined breeding colony, snakes are kept in different enclosures and are regrouped due to breeding purposes. Many adult snakes are singly housed in a terrarium or housed together with a breeding partner. Clutches often stay together in one enclosure for their 1st year of life.

**FIG 5:**
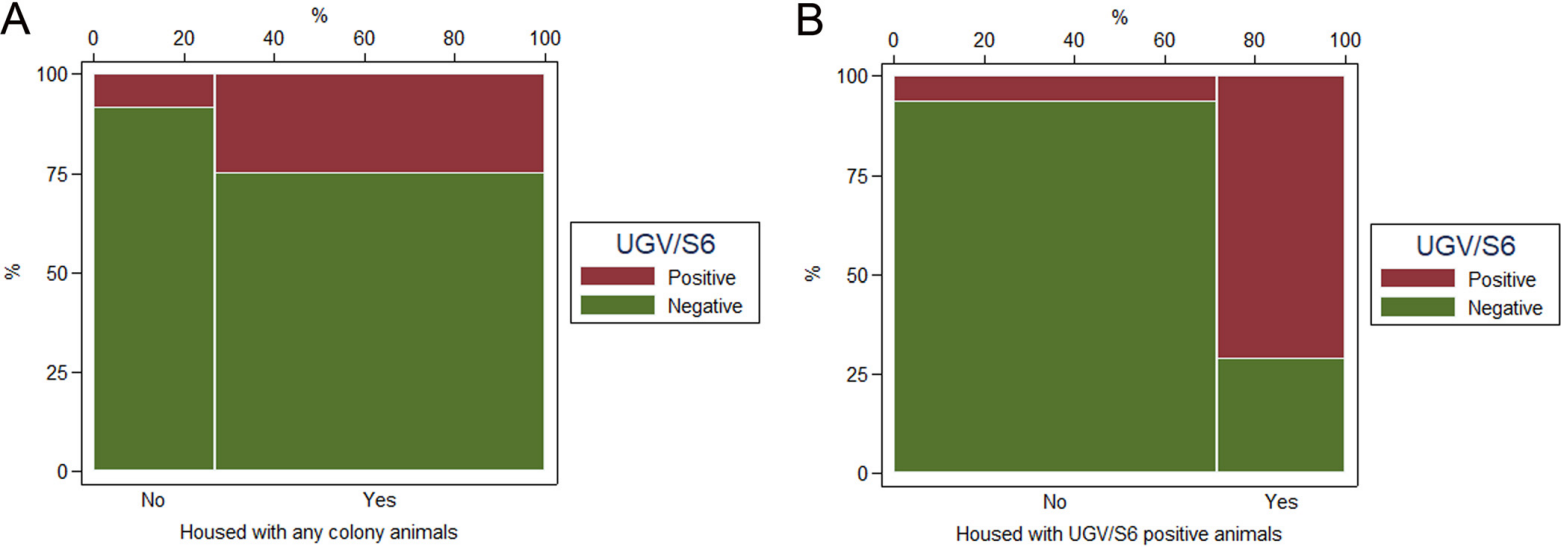
Mosaic plots illustrating the association between cohousing with other animals and reptarenavirus infection (determined with a qRT-PCR for UGV/S6 S segment). (A) Association between UGV/S6 infection status and cohousing in general. (B) Association between UGV/S6 infection status and cohousing with UGV/S6-positive animals.

Information on whether the snakes were housed with other snakes at any stage during their life was available for all 183 animals. The majority (134; 73.22%), 101 (75.37%) UGV/S6 negative and 33 (24.63%) UGV/S6 positive, were housed with other snakes. Of the remaining 49 (26.78%) animals, which were not housed with other snakes, 45 (91.84%) were UGV/S6 negative and 4 (8.16%) UGV/S6 positive. There is a positive association between cohousing and UGV/S6 status (χ^2^ = 6.029, *P* < 0.05) (Fig. 5A).

When the UGV/S6 status of the 134 cohabiting snakes was examined, 40 (29.85%) were found to be housed with UGV/S6-positive animals. Of these 40, 28 (70.00%) were UGV/S6 positive. Ninety-four (70.15%) were housed with UGV/S6-negative animals, and 5 (5.32%) of these were UGV/S6 positive. There is a positive association between positive UGV/S6 status and cohabitation with UGV/S6-positive animals (χ^2^ = 63.2425, *P* < 0.001) (Fig. 5B).

In the past, the population had been fluctuating consistently due to the selling and purchase of animals. It was therefore not possible to obtain information on the parents of all snakes. However, we could identify 24 animals for which we know the mothers and 25 for which we know the fathers. We also know from 69 snakes (23 UGV/S6 positive, 46 negative) that they were used for breeding, and 16 of these were housed with a UGV/S6-positive snake.

Specific information on parental animals and their offspring was available for five breeding events. We found different constellations of UGV/S6-negative and -positive parental animals and their offspring. For example, the UGV/S6 S-segment-positive parents (animals 20 and 58) had a clutch of which one UGV/S6 S-segment-positive male (animal 13; housed on its own) and 3 negative females (animals 92, 113, and 114; each housed with other, negative animals), aged 2 years and housed on their own, were examined in the present study. While the parental animals had two L segments (animal 20, male, ABV-3/L3 and TSMV-1/L7) or four L segments (animal 58, female, ABV-3/L3, TSMV-1/L7, KePV-1/L15, and KaBV-1), respectively, the only UGV/S6-positive offspring (animal 13) was male, was tested at 2 years of age, and carried only one L segment (ABV-3/L3). On another occasion the same female (animal 58) was mated with a UGV/S6-positive male (animal 56; with 2 L segments, ABV-3/L3 and TSMV-1/L7). Two female offspring (animals 109 and 110), 2 years of age and now singly housed, were available for the present study; both were UGV/S6 positive and harbored only the ABV-3/L3 L segment. The male snake (animal 20) was also mated with a UGV/S6 segment-negative female (animal 34); their offspring (3 female snakes, animals 82, 83, and 94, were available for the study) were negative at the time of sampling, at 2 years of age. A 2-year-old male snake, testing UGV/S6 negative, was also an offspring of the same male (animal 20); however, information on the mother was not available. Another breeding pair, animals 86 and 87, tested UGV/S6 negative in the present study. The entire clutch of 10 animals (animals 116 to 125, 1 year of age at testing) and a female from a previous clutch (animal 181) tested UGV/S6 positive. However, according to the breeder’s records, both parental animals had tested positive for reptarenavirus infection in a commercial laboratory approximately 2 years earlier.

In the majority of the offspring (14 animals), we identified one L segment (13 snakes with ABV-3/L3 and one snake [animal 120] with KePV-1/L15); 10 animals harbored two L segments, seven had three L segments, and six had four L segments.

All siblings in the younger clutch were IB positive.

## DISCUSSION

BIBD can cause severe losses in private and zoological boid snake collections, due to the recommendation for euthanasia (1). The present study aimed to establish a diagnostic workflow that would allow the screening of larger snake colonies not only for BIBD (both clinical and nonclinical) but also to identify the carriers of reptarenavirus infection. Such screening approaches would be essential to avoid and control the spread and/or introduction of reptarenaviruses and BIBD into, between, within, and from commercial breeding colonies. Reliable detection of reptarenavirus infection is challenging due to the large genetic variability among reptarenaviruses found in captive snakes, where coinfections are also common (3, 4, 8, 16, 18, 26, 27). Therefore, we reasoned that by identifying the reptarenavirome of a colony using metatranscriptomics, we could set up specific “personalized” tools to identify both the reptarenavirus carriers and the reptarenavirus-free animals in the colony. To test the approach, we studied 183 boa constrictor blood samples submitted for BIBD diagnosis from a large single breeding colony with a history of BIBD cases. By traditional blood smear analysis, we found a substantial proportion (15.3%) of the animals to demonstrate IBs in the blood cells; all of these were clinically healthy at the time of blood sampling, representing nonclinical BIBD cases. We selected seven animals with IBs and eight animals without any evidence of IB formation in blood cells for NGS. This approach identified the “colony-specific” reptarenavirome to comprise one S segment (UGV/S6) and three L segments (ABV-3/L3, TSMV-1/L7, and KePV-1/L15).

Others and we have shown that snakes with BIBD frequently carry several genetically variable reptarenavirus L and S segments (13, 16–18, 20). The studies have further indicated that the UGV/S6 S segment most commonly associates with IB formation and thereby BIBD in captive boas (16–18, 20). The fact that this was also the only S segment detected in the large colony investigated in the present study further supports the association between UGV/S6 S segment and BIBD. Based on the observations of one of the authors (U.H.), the BIBD phenotype has changed over the past decades, with nonclinical BIBD, i.e., the presence of IBs without overt clinical signs, becoming the rule, especially among boa constrictors. At the same time, the amount, size, and distribution of IBs within the affected organs appear comparable to those in the early days when clinical BIBD was common. In addition to diagnostics-related biases, i.e., more frequent testing and earlier diagnosis due to increased awareness among veterinarians and snake owners and breeders, the changes in BIBD phenotype could relate to adaptation of reptarenaviruses to boa constrictors or adaptation of the captive boa constrictors to reptarenavirus infection. On the adaptation front, the alteration of the virus would seem more likely and could, e.g., be mediated by the loss of the most virulent S segments from the circulating reptarenavirus S and L segment swarms. The host adaptation, albeit less likely, could relate to vertical transmission of the S and L segments in the captive populations and might be, e.g., mediated by immunotolerance toward reptarenaviruses.

Using an RT-PCR targeting the UGV/S6 S segment, we could confirm reptarenavirus viremia in all animals diagnosed as BIBD positive through blood smear analysis. Indeed, the detection of UGV/S6 S segment RNA was significantly associated with the presence of IBs. However, among the 155 snakes deemed BIBD negative based on the lack of IBs, the RT-PCR identified nine (5.8%) as reptarenavirus infected, highlighting the importance of molecular diagnostics for the screening of a given colony for BIBD/reptarenavirus infections. A previous study reported that the majority of reptarenavirus-infected, IB-positive snakes are clinically healthy (22). The present results provide further evidence of this, since the breeder reported that all animals appeared clinically healthy at the time of the blood sampling (personal communication). In recent years we have seen more often clinically healthy boa constrictors in good body condition which even produce BIBD-positive offspring (17), in contrast to the early phase of the disease when severe central nervous system (CNS) signs like “star gazing” and secondary infections with lethal outcomes dominated the clinical picture. The owner also decided to remove all IB-positive animals from the collection, and hence, retesting was not possible. Serial testing was suggested in another publication reporting on reptarenavirus infection and BIBD in pythons (6), although a recent study in boas found no increase in BIBD/reptarenavirus infection in serial testing as part of a long-time follow-up study (23). In the present study, retesting would have been very interesting, particularly in the case of the snakes with low UGV/S6 S segment RNA levels. However, we have anecdotal evidence from a few animals that transient reptarenavirus viremia might occur: one breeding pair that tested UGV/S6 negative and in which NGS had not identified any reptarenavirus in the blood (animals 86 and 87) had tested positive for reptarenavirus infection in a commercial lab around 2 years earlier. Vertical transmission from these animals to the offspring during a viremic phase of either parental animal would explain why we found all these animals to be UGV/S6 S segment positive. We also found out that one UGV/S6 S-segment-positive snake (animal 20; housed with another positive animal) had tested negative 3 years ago, after it had been imported from the United States. By the time it was tested as part of the present study, it had developed IBs and produced infected offspring; when the infection occurred cannot be clarified anymore, but since this snake’s reptarenavirome was identical to that of the colony, it is likely that the animal became infected during cohousing and breeding with an infected male (animal 58).

The earlier findings of BIBD association with the presence of the UGV/S6 S segment, together with the fact that the S segment encodes NP, i.e., the main protein component of the IBs, motivated us to study if UGV/S6 S segment RNA levels would correlate with the presence of IBs. Indeed, we found significantly higher UGV S segment RNA levels in IB-positive snakes than in the animals without IBs. However, the examination of population parameters showed neither an association of sex and age with the disease nor an association of UGV S segment RNA levels with age, which suggests that the time of infection and the duration of viremia might be more important factors in the development of IBs than the animals’ age. We also found a few outliers, i.e., one reptarenavirus-positive animal without IBs (animal 35) with a UGV/S6 S segment RNA level that would also be considered very high in snakes with IBs, followed by the siblings 109 and 110, and one IB-positive snake (animal 5) with a UGV/S6 S segment RNA level around the average of animals that did not show IBs. While the reasons behind the various S segment RNA levels could be many, one could speculate the number of L segments to be a contributing factor to both RNA levels and the presence of IBs. Also retesting animal 35, which had a high UGV/S6 S segment RNA level but no IBs, would have been interesting, as it might have represented a snake on the verge of developing BIBD.

Because reptarenavirus NP, encoded in the S segment (15), is the main protein component of the IBs (3, 4, 21, 22), we speculate that there is a causal relationship between the presence of IBs and the number of carried L segments. We hypothesize that the number of L segments contributes to the amount of UGV/S6 S segment RNA because the L segment encodes the RdRp that is responsible for RNA replication and transcription: the more RdRp, the more viral segments and also NP mRNA. This motivated us to first screen the UGV-positive blood samples for the presence of the three L segments that we had identified as part of the reptarenavirome, ABV-3/L3, TSMV-1/L7, and KePV-1/L15-like L segment RNAs. Indeed, based on the RT-PCR results the number of L segments detected in the animals was significantly associated with UGV/S6 S segment RNA levels. For individual L segments, however, the results varied. After these results, we screened 12 of the UGV/S6 S-segment-positive animals for the presence of additional L segments employing primers (18 different reptarenavirus L segments) used in our earlier studies (17). Some of these animals produced a PCR amplicon with SVaV-1 or -2/L12 L or KaBV-1 L segment-targeting primers. The screening of all UGV/S6 S-segment-positive animals revealed 8 animals to yield a positive PCR result with SVaV-1 or -2/L12 and 10 animals to yield a positive PCR result with KaBV-1 L segment-targeting primers. Sanger sequencing and BLAST analysis showed that two animals carried reptarenavirus L segments quite distant from both SVaV/L12 and KaBV, while the majority of animals found positive by SVaV-1 or -2/L12 or KaBV-1 L segment-targeting RT-PCR were confirmed to carry SVaV-like and/or KaBV-like L segments. While for ABV-3/L3 there was no significant association with the presence of IBs and/or UGV/S6 S segment RNA levels, there was a significant correlation with UGV/S6 S segment RNA levels for TSMV-1/L7, and with the presence of IBs for the KePV-1/L15 L segment. Interestingly, we also found a negative association between the detection of the KePV-1/L15 L segment and male animals; this cannot be readily explained. The detection of SVaV/L12 showed a negative association with the levels of UGV/S6 S segment RNA, and the detection of KaBV L segment was significantly associated with the detection of IBs. The screening and diagnosis of reptarenavirus infection in snakes are challenging due to the genetic diversity among reptarenaviruses. We have made attempts at generating a “pan-reptarenavirus” RT-PCR tool but have stumbled into sensitivity/specificity issues with these approaches. The screening approach described and tested here is based on identification of the reptarenavirus segments in a subpopulation of snakes of the breeding colony by NGS and *de novo* assembly. As demonstrated, we could detect several segments that allowed screening of the entire collection with RT-PCRs designed on the basis of the NGS results. We were able to identify the most common segments present in the collection; however, additional screening indicated that the analysis may have overlooked some reptarenavirus segments. The identification of the additional L segments did not affect the results of the initial screening in terms of presence or absence of reptarenaviruses. However, the result indicates that even the NGS-based approach might not be sufficient to detect less abundant segments in a collection, even though the approach apparently identified all reptarenavirus segments of the analyzed animals, as we could not detect any reads in the NGS data corresponding to SVaV-1/L12 or the KaBV-1 L segment. Although currently not yet feasible as an affordable routine approach, it would still represent a reliable, ultimately efficient tool to clear large, closed breeding colonies (e.g., zoological collections) from the infection. As we altogether identified only one S segment but five reptarenavirus L segments in the colony, our results further support the idea of using an S-segment-specific RT-PCR for screening purposes. An L-segment-based screening approach would, depending on the target segment, have yielded very different results. Whether the quantification of UGV/S6 S segment RNA levels does indeed allow discrimination between virus carriers and animals that have already developed the disease needs to be determined in further studies on more random animal cohorts. The effect of multiple L segments for the S segment RNA level merits further studies, as it could help to explain the role of “coinfection” (or coexistence of multiple S and L segments) in BIBD pathogenesis.

## MATERIALS AND METHODS

### Animals and samples.

The study was performed on blood smears and full blood samples from 183 *Boa constrictor imperator* snakes that had been submitted for diagnostic purposes (BIBD screening and detection of reptarenavirus infection in individual animals). The study population comprised 112 female and 71 male individuals, aged between 1 and 10 years. The animals originated from a commercial breeding colony that had previously experienced BIBD cases. The colony comprises more than 200 breeding snakes, representing both in-house-bred animals and snakes introduced from external sources to avoid inbreeding and to introduce new color morphs into the collection. The last introduction of an external animal had taken place approximately 2 years prior to the sampling. Like all prior external animals, it had been admitted into the colony after a 4-month period in a separated quarantine room. The husbandry conditions in the colony include an air temperature of 27°C with a local heating spot in each enclosure of 29 to 35°C, humidity of approximately 45% and 70 to 80% after water spraying, and a season-dependent light regime that makes use of artificial lightning. The offspring are housed together in caging rack systems, composed of shelves including several (disposable) plastic tubs, as clutches stay together for the first 2 weeks of life, until the first shedding, after which they are separated and housed individually. Exceptions are the snakes chosen for breeding; these are kept in breeding groups of up to two males and two females in one enclosure. However, the changing of enclosures is common for each breeding period due to specific breeding strategies. The staff is trained to work hygienically when feeding the animals and cleaning the enclosures, to minimize contamination and spread of potential infectious agents over several cages and rooms.

After completion of the molecular analyses and identification of infected snakes, the breeder was contacted and asked for information on the relationship between infected snakes and other snakes in the collection, i.e., the relationship as parental animals, as siblings/offspring, and as breeding partners with each other. Information about specific housing groups and shared enclosures of reptarenavirus-positive and -negative snakes was also provided.

From each animal, a blood sample was taken by cardiocentesis/caudal tail vein venipuncture by the veterinarian attending the colony, stored in EDTA tubes (microtube, 1.3 mL; K3 EDTA; Sarstedt AG & Co. KG, Germany), and shipped, accompanied by a CITES (Convention on International Trade in Endangered Species of Wild Fauna and Flora) export and import permit, to the Institute of Veterinary Pathology, Vetsuisse Faculty, University of Zurich, on dry ice where the samples were stored at −80°C until analyzed. In addition, two blood smears were prepared on glass slides on a sampling site (Menzel-Glaeser Superfrost microscope slides; Gerhard Menzel GmbH, Germany) and shipped alongside the blood samples.

Since the work was undertaken for diagnostic purposes, no ethical permission was required. The owner gave full permission for the scientific use of the data obtained from the animals.

### Cytological examination.

For each animal, one blood smear was routinely stained with May-Grünwald-Giemsa stain and examined independently by two examiners, using a light microscope at a 400-fold magnification (sigmoidal screening, approximately 15 to 20 min per slide) for the detection of inclusion bodies (IBs) in blood cells, an approach applied in previous studies by our group (10, 11, 13, 17, 20).

### RNA extraction and cDNA synthesis.

RNA was extracted from 100 μL of each EDTA blood sample as previously described (17), including a mechanical homogenization step using a Retsch MM300 TissueLyser (Qiagen) for 2 min at high frequency (30 Hz) as applied in a previous study (20).

The amount of RNA in each sample was measured with a Qubit fluorometer and RNA BR assay kit (20- to 1,000-ng range) or RNA HS assay kit (5- to 100-ng range) (Thermo Fisher Scientific, Invitrogen). The cDNA syntheses were performed with a Revert Aid RT kit (Thermo Fisher Scientific) with random hexamer primers following the manufacturer’s protocol. The input RNA for the reaction mixture was between 5.1 and 100 ng/μL, and 5 μL of the obtained RNA was used in the cDNA synthesis. For four samples with RNA concentrations of ≤10 ng/μL, 11 μL of the obtained RNA was used.

### NGS.

Next-generation sequencing (NGS) and *de novo* assembly were undertaken on blood samples from eight IB-positive and seven IB-negative (no evidence of IBs in blood cells) snakes to identify the reptarenavirome of the breeding colony. The NGS library preparation in brief was as follows: after DNase I (Thermo Fisher Scientific) treatment of the purified RNAs and repurification using the GeneJET RNA purification kit (Thermo Fisher Scientific), further cleaning of the RNA was performed using the Ribo-Zero Gold rRNA removal kit for epidemiology (Illumina) according to the manufacturer’s protocol. The indexed NGS libraries were prepared using the NEBNext Ultra RNA library preparation kit and the NEBNext Library Quant kit for Illumina (all New England Biolabs) quantification, as previously reported (17). By using a MiSeq reagent kit (version 3; Illumina) 300-bp paired-end reads were sequenced on an Illumina MiSeq system (Illumina).

Removal of reads matching the host genome (known snake genome [Python bivittatus]; https://ftp.ncbi.nlm.nih.gov/genomes/refseq/vertebrate_other/Python_bivittatus/all_assembly_versions/GCF_000186305.1_Python_molurus_bivittatus-5.0.2/) and *de novo* sequence assembly were performed on a CSC (IT Center for Science Ltd., Finland) Taito supercluster server using Lazypipe (version 1.1.0, https://www2.helsinki.fi/en/projects/lazypipe) (25). Reference assemblies were performed in Unipro UGENE version 38.1 (28) utilizing the Bowtie2 tool (29).

### TaqMan quantitative reverse transcription-PCR (qRT-PCR) for the detection of UGV.

Since NGS and *de novo* assembly identified only one S segment, the one of University of Giessen virus (UGV), in the examined samples, we made use of the qRT-PCR primers (forward, 5′-CAAGAAAAACCACACTGCACA-3′, and reverse, 5′-AACCTGTTGTGTTCAGTAGT-3′; Microsynth AG, Switzerland) from an earlier study (19) in combination with a new probe for the detection of different UGV S segment variants (UGV-1 to -4) (5′-6-FAM [carboxyfluorescein]-AATGATGTGTCCTGAGGAATTGATCTTCA-3′-TAMRA [6-carboxytetramethylrhodamine] [Metabion International AG, Germany]) to screen all 183 animals for reptarenavirus infection. *In vitro*-transcribed RNA, corresponding to nucleotides 567 to 1021 of the UGV-1 S segment (GenBank accession number NC_039005.1), including the qRT-PCR target sequence corresponding to nucleotides 718 to 851, was produced as described in reference 30. This control RNA served to generate a standard curve (10-fold dilution series covering the range of 10^9^ to 50 copies/reaction) for converting cycle threshold values to copy numbers and subsequent determination of the number of UGV S segments in the infected animals. For each sample, the number of S segments was normalized to the amount of RNA used in the reaction.

The qRT-PCRs were run in duplicates with 2.5 μL TaqMan Fast Virus 1-step master mix (Thermo Fisher Scientific, Applied Biosystems), 0.5 μM forward and reverse primer, 0.25 μM probe, and 2.5 μL of the RNA template in a 10-μL total reaction volume using the following cycling conditions: (i) holding stage (step 1, 5 min at 50°C; step 2, 20 s at 95°C); (ii) cycling stage (step 1, 3 s at 95°C; step 2, 30 s at 60°C). Cycling stage 2 was repeated 36 times. A 7500 real-time PCR system and software v2.0 (Thermo Fisher Scientific, Applied Biosystems) served for cycling and data analyses.

### RT-PCR.

The NGS and *de novo* assembly approach also identified three L segments (aurora borealis virus 3 [ABV-3], tavallinen suomalainen mies viruses 1 and 2 [TSMV-1 and -2], and keijut pohjoismaissa virus 1 [KePV-1]). We analyzed the UGV S-segment-positive samples for the presence of the above-described segments with the primers and protocols employed in our earlier study (17). A subset (*n* = 12) of the UGV S-segment-positive samples were also tested for other L segments (UGV-1 to -3, ABV-1 and -4, University of Helsinki viruses 1 to 4 [UHV-1 to -4], suri vanera viruses 1 and 2 [SVaV-1 and -2], Hans Kompis virus 1 [HKV-1], HKV-like virus, ROUT virus [ROUTV], Golden Gate virus [GGV], CAS virus [CASV], bis spoeter virus 1 [BSV-1], gruetzi mitenand virus 1 [GMV-1], kuka mitä häh virus 1 [KMHV-1], and Kaltenbach virus 1 [KaBV-1]), using the primers and the protocols described in reference 17. The initial screening identified SVaV-1, SVaV-2, and KaBV-1 L segment-targeting primers as producing a PCR product in some of the samples included in the initial screen, after which all UGV/S6 S-segment-positive samples were tested with these primers. The L segment PCR amplicons were analyzed by standard agarose gel electrophoresis (gel strength, 2%; GeneRuler 100-bp DNA ladder [Thermo Fisher Scientific], Tris-acetate-EDTA [TAE] buffer, GelRed nucleic acid gel stain [Biotium, Inc., USA]), and the results were recorded with the UVP BioCoc-It imaging system (Thermo Fisher Scientific). Sanger sequencing (by Microsynth AG, Switzerland) served to confirm the presence of L segments in PCR products with low L segment levels and those obtained by reverse transcription-PCRs (RT-PCRs) with SVaV-1, SVaV-2, and KaBV-1 L segment-targeting primers.

### Statistical analyses.

Statistical analyses were performed to examine the possible association of UGV S segment RNA level and other measured and population parameters (age, sex, and housing).

A Shapiro-Wilk test was performed and indicated that the distribution of the UGV S segment RNA level departed significantly from normality (*W* = 0.8140, *P* value of <0.001). Square root transformation was used to achieve normality (*W* = 0.943, *P* = 0.056). The associations of UGV S segment RNA levels and the detection of population parameters, BIBD, and reptarenavirus L segments was examined at univariate level using the *t* test for all binary variables. Where stratified analysis was used, pairwise mean comparisons were performed using the Bonferroni correction. Linear regression was used to examine the association between the level of UGV S segment RNA and age, controlling for the presence of IBs. Associations between sex, BIBD, and reptarenavirus L segments and UGV/S6 status and housing records were examined using χ^2^ tests, while for the associations between age and binary variables, the Wilcoxon rank sum (Mann-Whitney) test was employed.

Results were analyzed using Stat13 (Stata statistical software: release 13, 2013; StataCorp LLC, College Station, TX).

### Data availability.

The raw NGS data have been deposited in SRA under BioProject accession number PRJNA966910.
