# Supplementary material for: Reptarenavirus S Segment RNA Levels Correlate with the Presence of Inclusion Bodies and the Number of L Segments in Snakes with Reptarenavirus Infection—Lessons Learned from a Large Breeding Colony
Source: Microbiol Spectr. 2023 May 22;11(3):e05065-22. doi: 10.1128/spectrum.05065-22 (PMC10269766; doi:10.1128/spectrum.05065-22)
Supplement: Supplemental file 1 — Tables S1 to S3. Download spectrum.05065-22-s0001.pdf, PDF file, 0.8 MB [file spectrum.05065-22-s0001.pdf]

Supplementary Table S1. List of animals found to carry the UGV-1/S6 S segment, with age, sex, BIBD status (determined by the presence of cytoplasmic inclusion bodies in blood smears in a May-Grünwald-Giemsa stained blood smear), reptarenavirus L segments (determined by segment-specific RT-PCR) and viral loads (as UGV-1 copies/ng of total RNA) determined by quantitative RT-PCR. Samples are ordered according to the BIBD status and increasing viral loads.

Sample No	Age (years)	Sex	BIBD status	L segments			UGV-1 S segment
				ABV/L3	TMSV/L7	KePV/L15	Copy numbers
36	3	0.1	-	+	-	-	0.5667
175	3	1.0	-	+	-	-	1.9493
143	1	1.0	-	+	-	-	2.1256
22	1	1.0	-	+	-	-	4.3536
13	2	1.0	-	+	-	-	10.7903
183	4	0.1	-	+	-	-	141.1939
109	2	0.1	-	+	-	-	822156.4643
110	2	0.1	-	+	-	-	1770758.8406
35	3	1.0	-	+	+	-	5217808.0000
5	3	1.0	+	+	-	-	402.8487
21	1	1.0	+	-	+	-	108674.8966
20	4	1.0	+	+	+	-	275299.6720
19	9	0.1	+	+	+	-	334657.1091
118	1	0.1	+	+	-	+	348419.8867
123	1	1.0	+	+	-	+	427178.7933
125	1	1.0	+	+	-	+	526317.3455
117	1	0.1	+	+	-	+	543386.7586
124	1	1.0	+	+	-	+	578604.1728
88	NK	1.0	+	+	+	-	823066.4673
119	1	0.1	+	+	-	-	855826.6347
103	2	1.0	+	+	+	-	1053333.3760
181	7	1.0	+	+	+	-	1373491.8261
122	1	0.1	+	+	-	+	1430379.8306
97	2	0.1	+	+	+	-	1439513.0435
89	NK	0.1	+	+	+	-	2099785.3107
170	5	0.1	+	+	-	-	2241266.2430
120	1	0.1	+	-	-	+	2281565.6250
169	3	1.0	+	+	-	-	2853737.7718
180	2	0.1	+	+	-	-	3053403.7228
116	1	0.1	+	+	-	+	3223812.8814
57	2	1.0	+	+	+	-	4014397.0909
179	10	0.1	+	+	-	+	5026935.9543
58	9	0.1	+	+	+	+	5946793.6296
59	6	0.1	+	+	+	+	6250268.3796
44	NK	1.0	+	+	+	-	6269959.1429
121	1	0.1	+	+	-	+	6347669.1014
56	3	1.0	+	+	+	-	9695507.2370

Sex: 1.0 - male, 0.1 - female; BIBD status: + - BIBD positive, - - BIBD negative; NK – not known; Copy numbers - normalized number of UGV-1 S segment copies/ng of total RNA

Supplementary Table 2. Square root of UGV/S6 S segment RNA level of the examined snakes against the presence of inclusion bodies (BIBD) and the detection of ABV3/L3, TMSV-1/L7, KePV-1/L15, SVaV, and KaBV L segment by RT-PCR.

Square root of normalised RNA copies per ng

	positive		negative		test
	Mean (n)	95%CI	Mean (n)	95%CI	
BIBD	1369.887 (28)	1063.30 - 1676.48	504.727 (9)	-133.67 - 1143.123	t=-2.8225, df = 35, p<0.01
ABV	1173.121 (2)	868.84 - 1477.40	920.072 (35)	-6581.839 - 8421.983	t=-0.3936, df = 35, p=0.6963
TMSV	1573.677 (14)	1061.434 - 2085.919	907.300 (23)	569.830 - 1244.771	t=-2.3924, df = 35, p<0.05
KePV	1472.443 (12)	968.706 - 1976.179	1009.203 (25)	643.470 - 1374.936	t=-1.5377, df = 35, p=0.1331
SVaV	1731.796 (8)	998.7399 - 2464.853	1001.552 (29)	690.1186 - 1312.986	t =-2.2013, df=35, p<0.05
KaBV	1506.741 (10)	908.914 - 2104.567	1030.814 (27)	687.7282 - 1373.9	t = -1.4962 df=35, p = 0.1436

Supplementary Table 3. Pairwise comparison of Square root of normalised RNA copies per ng means presenting difference and 95% confidence intervals (Bonferroni adjusted) between paired BIBD and different L segments.

			IB pos Diff (95%CI) p value		IB neg Diff (95%CI) p value	
	L segment	Mean (n)	Lseg pos	Lseg neg	Lseg pos	Lseg neg
IB pos.	ABV pos.	1404.488 (26)				
	ABV neg.	920.072 (2)	899.7617 (268.0874 - 1531.436) p<0.01			
IB neg.	ABV pos.	504.7267 (9)	484.4164 (-714.1013 - 1682.934) p=0.417	415.3453 (-861.4673 - 1692.158) p=0.513		
	ABV neg.	(0)				
IB pos.	TMSV pos.	1519.017 (13)				
	TMSV neg.	1240.641 (15)	-765.2353 (-2337.272 - 806.8018) p=0.329			
IB neg.	TMSV pos.	2284.252 (1)	278.3756 (-295.6512 - 852.4023) p=0.331	-1043.611 2608.144 - 520.9224 p=0.184		
	TMSV neg.	282.286 (8)	1236.731 (556.019 - 1917.443) p<0.005	958.3554 (295.1566 - 1621.554) p<0.01	2001.966 (395.2222 - 3608.71) p<0.05	
IB pos.	KePV pos.	1472.443 (12)				
	KePV neg.	1292.97 (16)	179.4723 (-447.3007 - 806.2452) p=0.564			
IB neg.	KePV Pos.	(0)				
	KePV neg.	504.7267 (9)	967.7161 (243.981 - 1691.451) p<0.05	788.2438 (104.3785 - 1472.109) p<0.05		
IB pos.	SVaV pos.	1652.874 (7)				
	SVaV neg.	1275.558 (21)	377.316 (-280.0556 - 1034.688) p=0.251			
IB neg.	SVaV pos.	2284.252 (1)	-631.378 (-2241.603 - 978.8468) p=0.431	-1008.694 (-2550.367 - 532.9789) p=0.192		
	SVaV neg.	282.286 (8)	1370.588 (591.0414 - 2150.135) p<0.005	993.2722 (367.4731 - 1619.071) p<0.005	2001.966 (404.3708 - 3599.562) p<0.05	
IB pos.	KaBV pos.	1506.741 (10)				
	KaBV neg.	1293.858 (18)	212.883 (-433.4161 - 859.182) p=0.508			
IB neg.	KaBV pos.	(0)				
	KaBV neg.	504.7267 (9)	1002.014 (249.0982 - 1754.929) p<0.05	789.1309 (120.1478 - 1458.114) p<0.05		
